# Propagation of antibiotic resistance genes during anaerobic digestion of thermally hydrolyzed sludge and their correlation with extracellular polymeric substances

**DOI:** 10.1038/s41598-022-10764-1

**Published:** 2022-04-25

**Authors:** Nervana Haffiez, Seyed Mohammad Mirsoleimani Azizi, Basem S. Zakaria, Bipro Ranjan Dhar

**Affiliations:** Civil and Environmental Engineering, University of Alberta, 116 Street NW, Edmonton, AB T6G 1H9 Canada

**Keywords:** Environmental impact, Chemical engineering

## Abstract

The positive impact of the thermal hydrolysis process (THP) of sewage sludge on antibiotic resistance genes (ARGs) removal during anaerobic digestion (AD) has been reported in the literature. However, little information is available on how changes in different extracellular polymeric substances (EPS) due to THP can influence ARG propagation during AD. This study focused on systematically correlating EPS components and ARG abundance in AD of sewage sludge pretreated with THP (80 °C, 110 °C, 140 °C, 170 °C). THP under different conditions improved sludge solubilization followed by improved methane yields in the biochemical methane potential (BMP) test. The highest methane yield of 275 ± 11.5 ml CH_4_/g COD was observed for THP-140 °C, which was 40.5 ± 2.5% higher than the control. Increasing THP operating temperatures showed a non-linear response of ARG propagation in AD due to the rebound effect. The highest ARGs removal in AD was achieved with THP at 140 °C. The multivariate analysis showed that EPS polysaccharides positively correlated with most ARGs and integrons, except for macrolides resistance genes. In contrast, EPS protein was only strongly correlated with β-lactam resistance genes. These results suggest that manipulating THP operating conditions targeting specific EPS components will be critical to effectively mitigating the dissemination of particular ARG types in AD.

## Introduction

Concerns regarding the release of antibiotics and subsequent dissemination of antibiotic resistance bacteria (ARB) and antibiotic resistance genes (ARGs) in natural and engineered ecosystems have emerged in recent years^1,2^. Notably, engineered ecosystems like wastewater treatment plants (WWTPs) are considered hotspots for ARGs propagation and transmission to the natural ecosystems^1,3^. Residual antibiotics in wastewater and sludge can stimulate resistance expansion by selective pressure on the resistant strains^3^. Through horizontal gene transfer (HGT), ARGs are spread among microbial communities via mobile genetic elements such as integrons^1,4^. In WWTPs, ARGs have been detected in all stages of wastewater and sludge treatment processes^2,5^. Remarkably, the land application of sludge from WWTPs has been identified as a significant route for ARGs transmission to the natural ecosystem, posing severe threats to the environment and human health^1,6^.

Anaerobic digestion (AD) is a widely applied approach for sludge processing in WWTPs, which focuses on three critical aspects: energy recovery, sludge reduction, and pathogen removal prior to land application or disposal of sludge^7,8^. Based on several reports, the conventional mesophilic AD is often ineffective in adequately removing ARGs and encourages HGT^9–11^. For instance, Yang et al. reported an increase in the abundance of various ARGs (e.g., *sul*1, *sul*2, *drf*A7, and *qnr*S*)* after mesophilic AD of swine manure^9^. Also, several mesophilic AD studies reported increased tetracycline resistance genes in digestate^10,12,13^. Thus, considerable momentum has been gained towards exploring effective remediation methods for sufficient ARG removal. Notably, the effects of the thermal hydrolysis process (THP) on ARG removal have received specific attention, which has been implemented in many WWTPs as a pretreatment method for the sludge solubilization before the AD^2,8,12,14^. The application of THP can overcome the rate-limiting hydrolysis during AD, leading to efficient biogas production. Moreover, THP improves sludge dewaterability and provides pathogens sterilization^2,7,15,16^. THP is often considered more economically attractive than other pretreatment methods (e.g., mechanical, chemical methods) due to energy recovery as heat from pretreated sludge^8^. To date, a few studies demonstrated that THP could provide removal of ARGs before AD^2,12,14,17^. It has been suggested that applying high temperature and pressure during THP can result in disintegration of cell walls and hydrolytic destruction of ARGs^2,18^. Furthermore, THP was found to be effective in degrading several antibiotics in AD feedstock, such as tetracycline, lincosamides, and macrolides, thereby reducing the selection pressure for ARG propagation during AD^18,19^. However, it has also been reported that some ARGs removed during THP could still rebound in the subsequent AD^2,12,14^. Despite such rebounding, THP-AD could provide better ARG removal than conventional AD without THP^2,12,14^.

A critical feature of sewage sludge is the presence of extracellular polymeric substances (EPS), consisting of various organic biopolymers such as carbohydrates, proteins, and humic substances produced by microorganisms^20–22^. High ARG abundance was found in the EPS matrix in aerobic activated sludge flocs in WWTPs^23^, which could be attributed to the high DNA adsorption ability of EPS^24^. Although extracellular ARGs can be primarily restricted in the EPS layer, they play a critical role in the ARG dissemination via HGT^23^. Notably, EPS-associated ARGs have exhibited higher transformation abilities than cell-free ARGs in the activated sludge process^23^. Another report suggested a positive correlation between ARGs abundances in membrane foulant and EPS content (protein and polysaccharide) in an anoxic/aerobic membrane bioreactor^25^. To the best of the authors’ knowledge, related information for AD or THP-AD has not been examined or reported in the literature. Particularly, THP of sludge prior to AD can significantly influence the solubilization of sludge EPS^8,26,27^. Under different THP operating temperatures, EPS solubilization may respond differently^28,29^. However, the correlation between THP operating temperatures, EPS composition, and ARGs has not been investigated for AD.

Based on the aforementioned research gaps, this study investigated the fate of ARGs in sewage sludge and their correlation with EPS in THP-AD. First, the effects of low and high-temperature THP (80–170 °C) on EPS characteristics and functional groups of the sludge were studied. Second, the quantitative and qualitative characteristics of microbial communities were analyzed. Third, EPS composition and ARGs abundances before and after AD were explored. To the best of the authors’ knowledge, this study is the first to provide new insight towards a better understanding of EPS composition and the ARG abundances in AD integrated with THP.

## Material and methods

### Sludge and inoculum

Primary sludge, waste activated sludge, and anaerobically digested sludge were collected from the Gold Bar Wastewater Treatment Plant (Edmonton, Alberta, Canada) and stored at 4 °C before use. The primary sludge (i.e., settled solids from primary clarifier) was mixed with waste activated sludge at a volume ratio of 1:1 and used for the experiment. The detailed characteristics of sludge and inoculum are provided in Table 1.

**Table 1:** Characteristics of substrates and inoculum.

Parameters	Inoculum		Substrate	
	Digested sludge	PS^a^	TWAS^b^	PS + TWAS^c^
TSS (mg/L)	16,625 ± 125	33,875 ± 125	26,500 ± 250	30,188 ± 63
VSS (mg/L)	16,625 ± 1,250	30,500 ± 1,500	24,875 ± 375	30,188 ± 937
TCOD (mg/L)	24,188 ± 18	50,126 ± 750	39,385 ± 345	44,756 ± 210
SCOD (mg/L)	3280 ± 97	5152 ± 75	2047 ± 51	3600 ± 13
TVFA (mg COD/L)	125 ± 2	1,125 ± 13	103 ± 3	614 ± 4
TAN (mg/L)	1244 ± 57	79.45 ± 1.9	199 ± 1.5	139.2 ± 1.4
pH	7.50 ± 0.01	6.4 ± 0.01	6.6 ± 0.01	7.4 ± 0.04

^a^Primary sludge (PS).

^b^Thickened waste activated sludge (TWAS).

^c^Mixture of PS and TWAS (volume ratio of 1:1).

### Thermal hydrolysis and biochemical methane potential (BMP) test

2 L bench-scale hydrothermal reactor (Parr 4848, Parr Instrument Company, Moline, IL, USA) was used for thermal hydrolysis of sludge at four different temperatures (80 °C, 110 °C, 140 °C, 170 °C) for 60 min exposure time. This exposure time is within the range reported in the literature^7,30^. For each experimental condition, 500 mL of feedstock (mixture of primary sludge and waste activated sludge) was fed to the hydrothermal reactor. The detailed operating protocol has been described elsewhere^27^.

The biomethane potential of raw and pretreated sludge was appraised with the BMP test. The BMP tests were performed with glass anaerobic bioreactors (working volume of 300 mL) equipped with mechanical agitators and electric motors (ISES-Canada, Vaughan, ON, Canada). The feedstock and inoculum volumes were used based on the food to microorganism ratio (F/M) of 2 (g of total chemical oxygen demand (TCOD) of sludge/g of volatile suspended solids (VSS) of inoculum). Furthermore, a blank test (inoculum + deionized water) was performed to evaluate methane production from the inoculum. Before start-up, the reactors were purged with nitrogen gas for 3 min and then placed in water baths at 37 ± 2 °C. The liquid was continuously mixed at 300 rpm. All tests were conducted in triplicate. The BMP tests were operated in a batch mode for 38 days, and the samples were taken before (day 0) and after the BMP tests (day 38) for analyses. Methane production was monitored daily using gas bags connected to sequestration bottles for capturing acidic gases (e.g., CO_2_, H_2_S). These bottles contained 3 M NaOH solution and a thymolphthalein indicator^31^. The methane gas volume was measured by a frictionless glass syringe.

### Analytical methods

TCOD, soluble chemical oxygen demand (SCOD), and total ammonia nitrogen (TAN) concentrations were measured with Hach reagent kits (Hach Co., Loveland, Colorado, USA) using UV-spectrophotometer (Model DR 3900, HACH, Germany). For SCOD and TAN, the samples were filtered using 0.45 µm membrane syringe filters. Total suspended solids (TSS) and VSS were measured according to the standard method^32^. The free ammonia nitrogen (FAN) concentrations were calculated according to the literature^33^. Ion chromatography (DionexTM ICS-2100, Thermos Scientific, USA) equipped with an electrochemical detector and microbore AS19, 2 mm column was used for volatile fatty acids (VFAs) concentrations measurement; the samples were filtered using 0.2 µm membrane syringe filters. A bench-top pH meter (AR15 pH meter, Fisher Scientific, Pittsburgh, PA) was used for pH measurement. The student's paired t-test was performed to manifest the statistical difference between the results obtained from different conditions using Microsoft Excel. Fourier-transform infrared spectroscopy (FTIR) analysis was performed as previously described in the literature^27^.

### Microbial community and DNA extraction

For microbial community analysis, digested sludge samples were collected after the BMP test on day 38, followed by centrifugation at 5000 rpm for 15 min, then 0.5 g of the pellet was taken for the DNA extraction. The genomic DNA extraction was accomplished by PowerSoil DNA Isolation Kit (MoBio Laboratories, Carlsbad, USA) according to the manufacturer’s instructions. For sequencing, the extracted DNA samples were stored at − 70 °C. The purity and concentration of DNA were detected by using the Nanodrop spectrophotometer (Model 2000C, Thermo Scientific, USA). The universal primer set 515F/806R has been used to target 16S rRNA using Illumina Miseq sequencing (Table S1). For microbial diversity evaluation, the nucleotides sequence reads were stored out by using a data analysis pipeline. A denoising and chimera detection steps were carried out to remove short sequences, chimeric sequences, and noisy reads. After that, each sample was run using the analysis pipeline to determine the taxonomic information for each component in the sample. Quantitative Insights Into Microbial Ecology (QIIME) pipeline (QIIME2, Version 2021.2) was used to analyze microbial communities’ taxonomy according to Zakaria et al.^34^.

### Quantification of ARGs

Quantitative polymerase chain reaction (qPCR) was used for quantifying thirteen frequently detected ARGs including 7 tetracycline resistance genes (*tet*A, *tet*B, *tet*C, *tet*W, *tet*M, *tet*Q, *tet*X), 2 sulfonamide resistance genes (*sul*1, *sul*2), 2 macrolide resistance genes (*erm*B, *erm*C) and 2 ß-lactam resistance genes (*bla*_AOX_, *bla*_TEM_). In addition, integrons (*intl1*, *intl2*) and 16S rRNA were also quantified. The primers of the selected genes are provided in Table S1. QuantiFast SYBR Green PCR Kit (Qiagen, CA) was used for the preparation of qPCR mixtures in 25 µL reactions as following: 2 µL of the DNA template, 12.5 µL 2 × master mix, 2.5 µL forward and reverse specific primer, and 5.5 µL nuclease-free water. Then, the CFX 96 real-time PCR system with a C1000 Thermal Cycler (Bio-Rad, USA) was used for the quantification process according to the QuantiFast SYBR Green PCR Kit’s protocol. The PCR initial heat activation cycle at 95 °C for 5 min, 35 cycles at 95 °C for 10 s and 60 °C for 30 s, and one cycle at 40 °C for 30 s. All samples were run in triplicate.

### EPS characterization

The EPS extraction was carried out by the heating method due to its high performance reported in the literature^35^. The biomass samples were centrifuged at 3000 × g for 15 min at 21 °C. Then, the supernatant was removed, and the pellet was washed with 0.1 M PBS (pH 7.4) three times. After washing, pellets were collected for cell lysis rate examination by Glucose-6-Phosphate Dehydrogenase kit (Sigma-Aldrich, USA). The details of EPS extraction and analytical methods were performed as previously described in the literature^34^. Carbohydrates were measured using the phenol–sulfuric acid method using glucose as a standard; details could be found in the literature^34^. After mixing 2 mL and 5 mL of EPS and concentrated H_2_SO_4_, respectively, 0.05 mL of 80 wt.% phenol was added. Then, the sample was left at room temperature for 10 min before shaking and incubating at 30 °C for 20 min. After cooling down to room temperature and using the UV-spectrophotometer (Model DR 3900, HACH, Germany), the absorbance measured 490 nm. The EPS protein content was determined by using the Pierce Modified Lowry Protein Assay Kit (Thermo Fisher, USA) according to the manufacturer’s instructions.

### Multivariate analysis

Using Spearman's rank correlation, ARG abundance, EPS components, and microbial communities were correlated with different operating conditions^36^. The correlation coefficient values ranged from − 1 to + 1, the higher positive values indicating stronger correlation and lower negative values indicating weak correlation. The alpha value for the correlation confidence intervals was set up as 0.05. Correlation analyses were visualized in principal component analysis (PCA) generated by JMP software (SAS Institute Inc., Cary, NC, US) and R software (RStudio v.1.4.1103).

### Kinetic analysis

The methanogenesis kinetics for different conditions were assessed based on the BMP test experimental data using the modified Gompertz model. The detailed methodology for estimation of kinetic parameters can be found elsewhere^27,37^.

## Results and discussion

### Effect of THP on sludge solubilization

**Figure 1:**
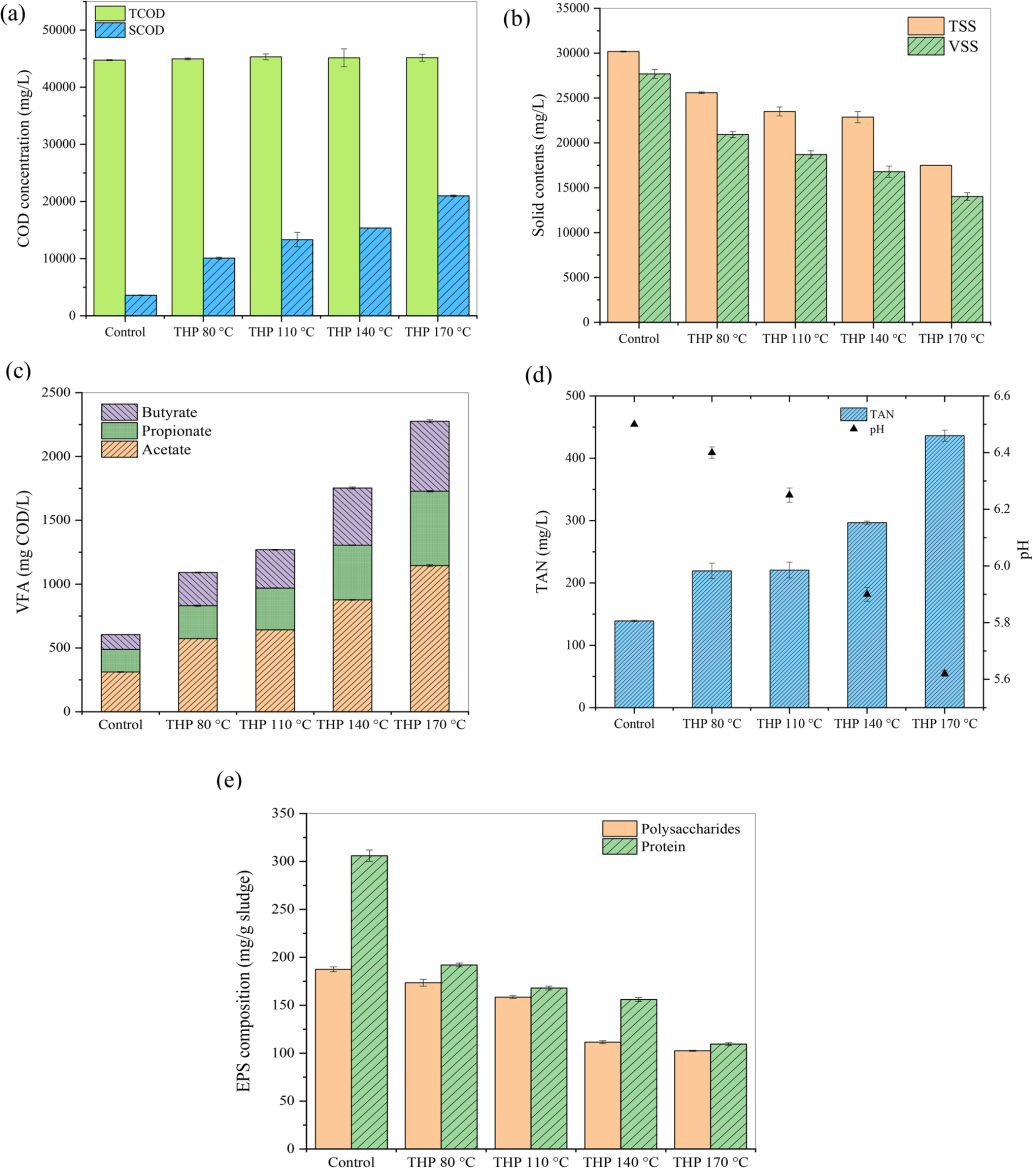
Effects of THP on (**a**) TCOD and SCOD, (**b**) TSS and VSS, (**c**) VFAs, (**d**) TAN, (**e**) EPS concentrations.

As shown in Fig. 1a, an increase in SCOD levels points to the disintegration of particulate biopolymers to soluble monomers and the release of the water-soluble components due to THP ^7^. Notably, SCOD concentrations increased (*p* = 0.00004) from 3599 ± 12 mg/L for the raw sludge to 20,998 ± 132 mg/L after THP at 170 °C (Fig. 1a). However, TCOD concentrations remained unchanged, indicating no volatilization of organics occurred during THP. Thus, SCOD/TCOD ratios in pretreated sludge dramatically increased compared to the raw sludge (Fig. S1). After THP, TSS and VSS concentrations also considerably decreased in the pretreated sludge (Fig. 1b). All pretreatment conditions significantly decreased VSS/TSS ratios (Fig. S1). The VSS reductions were 24.4%, 32.5%, 39.3%, and 49.3% for THP at 80 °C, 110 °C, 140 °C, and 170 °C, respectively. Thus, noticeably, increasing THP operating temperature increased the solubilization of particulate organics linearly.

As shown in Fig. 1c, TVFA concentration increased from 604 ± 2.8 mg COD/L (raw sludge) to 1,091 ± 11.3 (*p* = 0.003), 1,269 ± 5 (*p* = 0.001), 1,752 ± 7.3 (*p* = 0.005), and 2,275 ± 14.9 (*p* = 0.003) mg COD/L for THP at 80 °C, 110 °C, 140 °C, and 170 °C, respectively. Similar to SCOD, VFA concentrations also increased with increasing THP operating temperature. These results agree with previous reports on increasing organics and suspended solids solubilization with increasing THP temperatures in different ranges (50–170 °C)^26–28^. As suggested in the literature, enhanced hydrolysis could ultimately increase the downstream acidogenesis process for the production of VFAs^38,39^.

Compared to the raw sludge, TAN concentrations increased in all pretreated samples (Fig. 1d). Notably, TAN concentration reached up to 436.3 ± 8.9 mg/L (*p* = 0.008) at 170 °C, while TAN concentration in raw sludge was 139.15 ± 1.2 mg/L. A significant increment of TAN concentrations is typically attributed to the hydrolysis of nitrogenous compounds, such as proteins^27^. Despite remarkable increments in TAN after THP, TAN levels in all the samples were lower than 440 mg/L, which was much lower than inhibitory TAN concentrations (4.2 g/L) previously reported for AD^40^. Due to the further hydrolysis during AD, TAN concentrations increased > 1,000 mg/L in digestate samples after the BMP test (Fig. S2b), with the highest concentration of 1446.05 ± 0.95 mg/L (*p* = 0.001) was observed for the digested THP sample at 170 °C. However, the digester operating conditions, methanogenic communities can be inhibited if FAN concentration is around 215–1450 mg/L ^41^. In both raw and pretreated samples, FAN concentrations were < 1 mg/L (Fig. S2a). Although FAN concentrations increased in all digestate samples, the pretreated digestate samples showed considerably lower FAN levels than the control. Notably, the highest FAN of 209 ± 0.77 mg/L was observed for the digested control sample, while the lowest concentration of 169 ± 0.13 mg/L (*p* = 0.004) was observed for the digested THP 140 °C. As the operating temperature was the same for all conditions, estimated FAN concentrations (Fig. S2c) were correlated with TAN and pH values (Fig. S2b).

### Changes in EPS and macromolecules

The changes in EPS were characterized in terms of polysaccharides and proteins as they are considered the most dominant EPS components in sludge^42^. As shown in Fig. 1e, polysaccharides and proteins contents in sludge decreased after the THP. Polysaccharide and protein contents in the THP-80ºC sample were 173.5 ± 3.5 and 306 ± 6 mg/g sludge, which is 7 and 37%, respectively, lower than the control (i.e., raw sludge). The highest reduction of polysaccharide and protein (45 and 64%, respectively) was observed for THP-170 °C. Noticeably, EPS contents decreased gradually with increasing THP temperature. Previous studies also reported that THP could disrupt the EPS network, releasing intra- and extracellular organics in the aqueous phase^20,43^. Furthermore, FTIR analysis of solids was carried out to identify the effects of THP on functional groups associated with macromolecular compounds (Fig. S3). FTIR results also confirmed solubilization of macromolecular organics after the THP. Moreover, the gradual decrease of the absorption peaks with increasing the THP operating temperature accentuated the relationship between THP operating temperatures and solubilization efficiencies.

Both protein and polysaccharide contents in the extracted EPS from the digestate (after BMP) from THP-110 °C and THP-140ºC were less than those in control, THP-80 °C, and THP-170 °C samples (Fig. S4). Interestingly, THP-110 °C and THP-140 °C also showed higher methane production than other conditions (discussed later). Notably, a dramatic shift in the EPS composition was observed for the digestate THP-170 °C sample. That might be attributed to the microorganisms' protection mechanism that involves polysaccharides secretion to form a protective layer against the recalcitrant or inhibitory compounds commonly formed at high temperatures^22,44^. Moreover, for the THP-170ºC sample, the increased EPS contents during AD and decreased methane generation (discussed later) suggest that THP at 170ºC might form some recalcitrant/inhibitory compounds. Increasing EPS in the form of proteins can have a positive impact, as proteins can act as electron shuttles due to the exoenzyme's existence, enhancing the extracellular electron transfer and improving AD performance^44^. However, there is no evidence of such a positive impact of EPS polysaccharides^44^.

### Methane production

**Figure 2:**
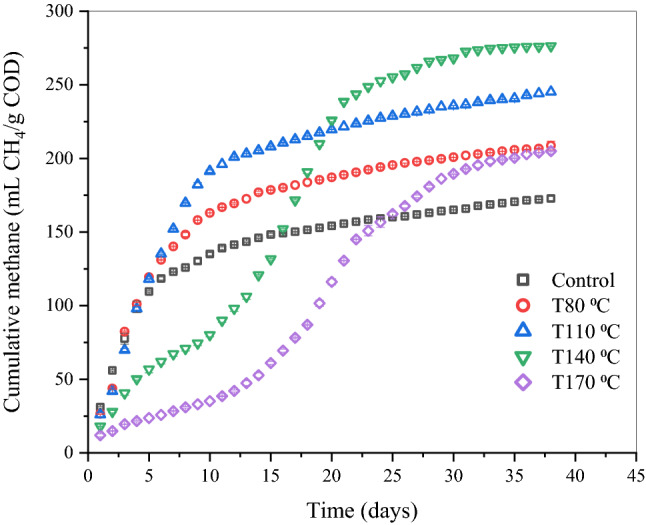
Cumulative methane production for raw and pretreated samples. Note: yields were calculated based on the initial COD of substrate.

As shown in Fig. 2, THP under different temperatures significantly improved the total cumulative methane yields than the control. For control, THP-80 °C, and THP-110 °C, methane production started immediately without any noticeable lag phases. In contrast, minor lag phases appeared for THP-140 °C and THP-170 °C. The estimated lag phases with the modified Gompertz model were also consistent with these experimental observations (Table S2). These results may attribute to the period that microorganisms need to adapt to the thermally pretreated sludge^37^. Particularly, high-temperature THP may release some refractory and inhibitory compounds that can extend the lag phases during AD^45^. Nonetheless, ultimately, all THP-treated samples led to higher total cumulative methane yields than the control. Despite higher lag phases than the control, THP-140 °C and THP-170 °C ultimately led to higher methane yields than the control, attributed to the higher maximum methane production rate than the control (see Table S2). The accumulated methane production increased by 20.6 ± 1.9%, 32.3 ± 1.7%, 40.5 ± 2.5% and 19.3 ± 0.2% for THP-80 °C, THP-110 °C, THP-140 °C, and THP-170 °C, respectively, compared to the control. Among the THP samples, the maximum methane yield (*p* = 0.03) of 275 ± 11.5 ml CH_4_/g COD was obtained for THP-140 °C, while the least methane yield (*p* = 0.07) of 203 ± 6.9 ml CH_4_/g COD was observed for THP-170 °C. Thus, the cumulative methane yields increased linearly with temperature increment except for THP-170 °C. The negative effect of THP at 170 °C on methanogenesis kinetics might attribute to recalcitrant compounds or toxic intermediates (e.g., melanoidins) formation^46^.

### Fate of ARGs

**Figure 3:**
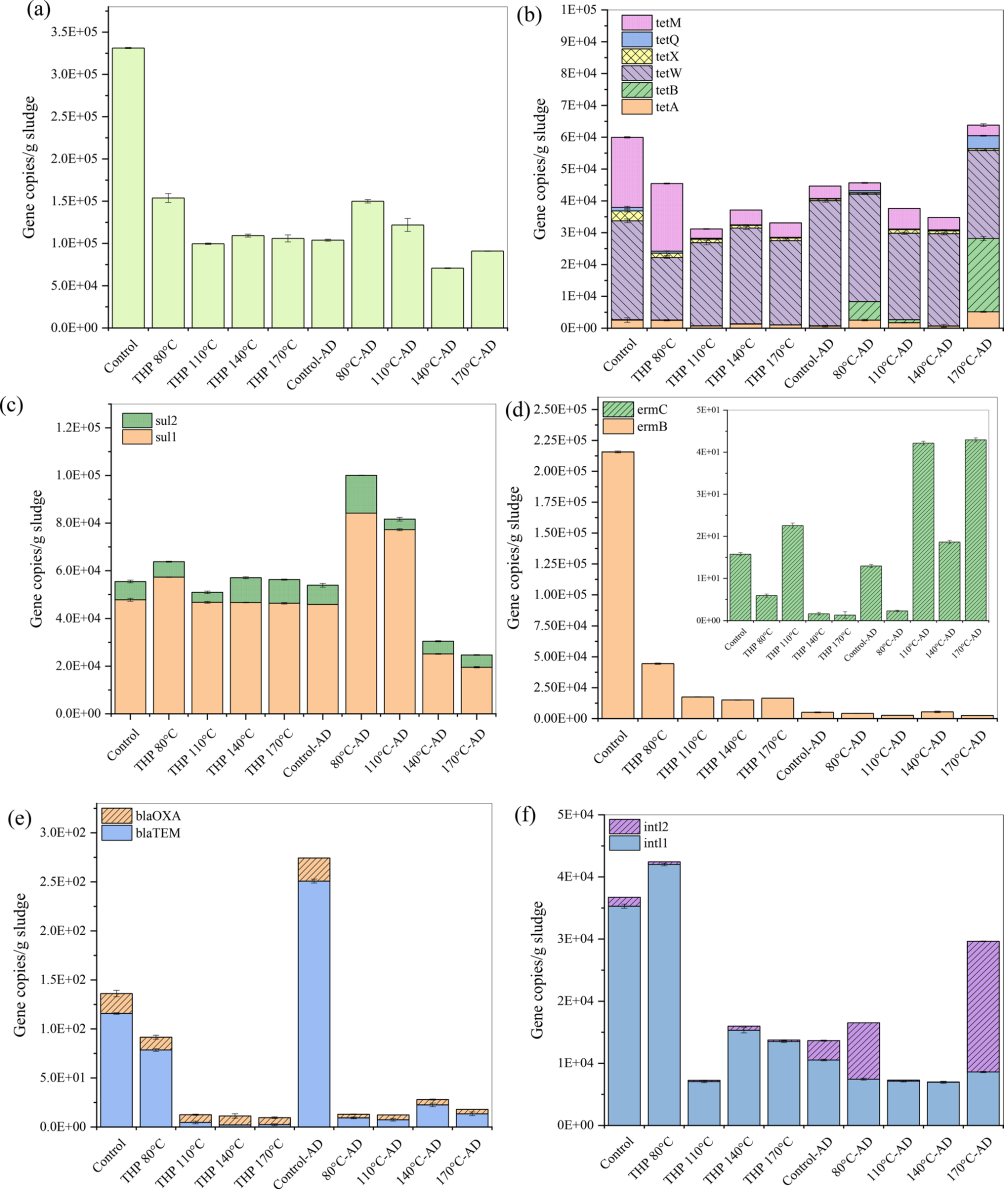
(**a**) Total ARGs, (**b**) tetracycline resistance genes, (**c**) sulfonamide resistance genes (**d**), macrolide resistance genes (**e**) β-lactam resistance genes, and (**f**) integrons in pretreated and digested sludge.

All targeted ARG subtypes were found in raw and pretreated sludge, as well as in the digestate after AD (Fig. 3). The absolute copy number of ARGs in the initial sludge sample was 3.31 × 10^5^ copies/g sludge (Fig. 3a). Compared to the raw sludge, a significant reduction (*p* = 0.000002—0.000009) of total ARGs was observed after THP. The ARGs copy number after THP at 80 °C, 110 °C, 140 °C, 170 °C were 1.54 × 10^5^, 9.96 × 10^4^, 1.09 × 10^5^, 1.06 × 10^5^, respectively (Fig. 3a). Thus, THP could remarkably reduce ARGs abundance prior to AD, while THP-110 °C provided the highest total ARGs removal (70%). The total ARGs also decreased in the subsequent AD except for the THP-110 °C sample (Fig. 3a). After AD, digestate from THP-140 °C and THP-170 °C showed lower ARG abundances than the digestate from control, while THP-140 °C was the most effective for overall ARG removal (79%) in the final digestate. Despite THP-110 °C being the most effective for ARG removal from before AD, the corresponding digestate sample showed an increase in ARG abundance (Fig. 3a). The increase in total ARGs for THP-110 °C sample indicates that rebounding of ARGs occurred during AD. Previous studies also reported similar ARG rebounding for thermally hydrolyzed sludge^8^.

As shown in Fig. 3b, c, various ARG subtypes (e.g., *tet*W*, tet*M*, tet*B*, tet*A, and *sul*1) might rebound during AD. Some potential ARG host microbes (e.g., *Treponema*^47^, *Pseudomonas*^47^, *Desulfotomaculum*^48^) can resist extreme environmental conditions (e.g., high temperature and pressure up) up to a certain limit during THP by forming endospores to cope with stressful conditions^49^. Thus, some host microbes might also exist in the pretreated sludge. Such a survival mechanism may happen under moderate temperature (110 °C) than high temperature (140 °C and 170 °C). When favorable conditions return, these endospores sprouts and the active bacterium is released to proliferate^50^. Thus, host microbes may proliferate in subsequent AD. Moreover, the possibility of residual DNA and horizontal gene transfer (HGT) may be a reason behind such rebounding ^12^. Noteworthy, the HGT is mediated by the MGEs, such as integrons (e.g., *intl*1, *intl*2) that control the DNA movement by encoding specific proteins^51^.

### Microbial quantity, diversity, and richness

**Figure 4:**
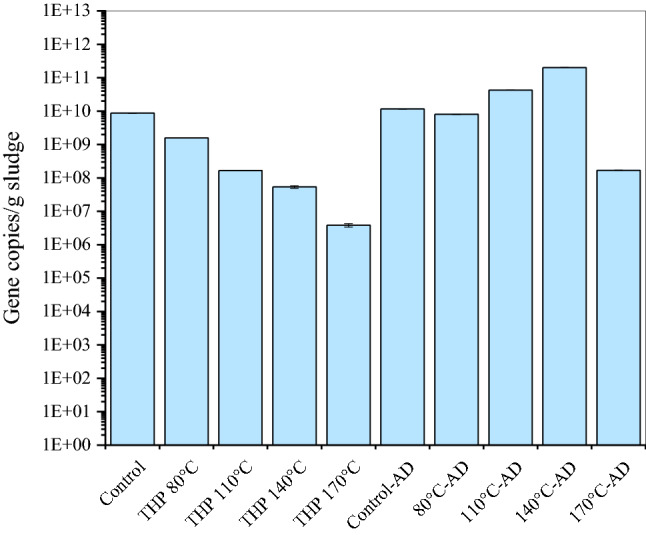
16S rRNA gene copies in raw, pretreated, and digestate sludge samples.

The quantitative PCR analysis was performed for the initial (inoculum + sludge) and final digestate. Due to the pretreatment, 16S rRNA gene copies remarkably decreased from 8.71 × 10^9^ gene copies/g sludge in control to as low as 3.84 × 10^6^ gene copies/g sludge for THP-170 °C (Fig. 4). However, 16S rRNA gene copies increased after AD. Notably, 16S rRNA gene copies gradually increased in digestate samples from THP-80 °C to THP-140 °C. The solubilization of organics via THP led to the proliferation of microbial communities. In contrast, the formation of recalcitrant or toxic compounds ^46^ at 170 °C might decrease microbial propagation.

The estimated alpha diversity indices were provided in Table S3. Compared to the control, all the indices were decreased after the THP except for the Chao1 and OTUs for THP-80 °C. The highest reduction in the microbial alpha diversity was observed for THP-170 °C. For instance, Chao1, Shannon, Pielou, and observed OTUSs were reduced from 171, 6.4, 0.86, and 170 to 95, 5.1, 0.79, and 95, respectively. Thus, THP could mostly reduce microbial community diversity and richness. This result agrees with previous studies that reported that temperature is the major factor affecting microbial alpha diversity^52^. Compared to the control, digestate for THP samples exhibited higher microbial diversity and richness. This might attribute to the enhanced sludge solubilization due to THP, which subsequently enhanced microbial diversity and richness during AD^53^.

### Bacterial community

Among the pretreated digested samples, dominant bacterial phyla were WWE1, Firmicutes, Chloroflexi, Bacteroidetes, and Proteobacteria (Fig. S5). Notably, members of WWE1 were the most dominant in all samples. They are known for the fermentation of sugars in AD^54^. Firmicutes are syntrophic bacteria involved in VFAs degradation^55^. Compared to the control, their relative abundance increased in digested THP samples. Chloroflexi species are known to hydrolyze carbohydrates^55^. Their relative abundance in control (21%) was higher than all digested THP samples. Bacteroidetes and Proteobacteria can degrade various organics, including cellulose and proteins^56^. Their relative abundances were higher in the digested THP samples than the control. Notably, their highest abundance was observed for the digested THP-170 °C sample (12 and 13%, respectively). Bacteroidetes and Proteobacteria are known as potential carriers of tetracycline resistance genes and other ARGs in general^57–59^. Thus, the highest abundance of tetracycline resistance genes observed in digestate of THP-170 °C sample was consistent with their high abundance.

**Figure 5:**
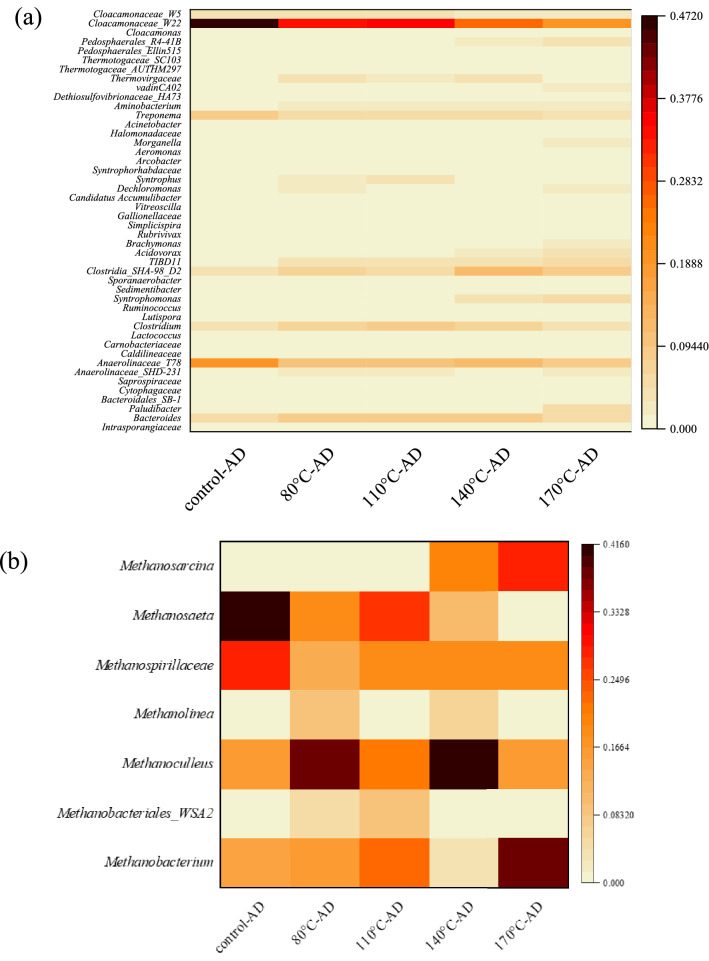
Relative abundance of (**a**) bacterial, (**b**) archaeal communities at the genus level.

At the genus level (Fig. 5a), the most dominant genera were *W22* (family *Cloacamonaceae*) and *T78* (family *Anaerolinaceae*) in all samples. Their highest abundances were observed in the control (47% and 20%, respectively), while both showed a remarkable decrease in the digested THP samples. The members belonging to *W22* (family *Cloacamonaceae*) were reported as syntrophic VFAs oxidizers^60^. Moreover, their potential roles in hydrolysis and acidogenesis have also been suggested^61^. The genus *T78* can contribute to hydrolysis/acidification, including carbohydrates and oil organics degradation^55,62^.

Like control, *W22* was still the most dominant genus (20–35%) in digested THP samples. However, their relative abundances noticeably decreased for higher THP operating temperatures (140–170 °C). Other dominant bacterial genera in digested THP samples include *Bacteroides* (6–8%)*, T78* (9–12%)*, Clostridium* (5–9%)*,* and *Treponema* (4–6%)*.* Members belonging to the genus *Clostridium* and *Bacteroides* are obligate anaerobes and can contribute to the fermentation of organics in AD^63,64^. Among all digested THP samples, the lowest relative abundances of these bacterial genera were observed for THP-170 °C. However, the abundance of *Syntrophomonas* and *Acidovorax* increased for this condition. Thus, these results indicate that increasing temperature led to distinct differences in bacterial communities.

### Archaeal community

Figure 5b shows the relative abundances of archaeal communities. The digested control sample was dominated by the genus *Methanosaeta* (41%), the family *Methanospirillaceae* (29%)*,* followed by genera *Methanoculleus* (16%), and *Methanobacterium* (14%)*.* The relative abundance of acetoclastic *Methanosaeta*^65^ was reduced in all digested THP samples. In contrast, various known hydrogenotrophic methanogens (*Methanoculleus*, *Methanospirillaceae,* and *Methanobacterium*) were dominant in these samples. *Methanoculleus* was the most prevalent in the digested THP-140 °C sample (41%). Also, hydrogenotrophic *Methanospirillaceae* (19%) and metabolically versatile *Methanosarcina* (20%) were dominant in this sample. The digested THP-170 °C sample was dominated by *Methanosarcina* (28%), and *Methanobacterium* (38%). Among the digested THP samples, the relative abundances of acetoclastic *Methanosaeta* species were higher in the digested THP-80 °C and THP-110 °C samples. Hydrogenotrophic methanogens usually have a higher ability to resist environmental changes than acetoclastic methanogens^66^. Thus, it appeared that high-temperature THP (140 and 170 °C) might have more pronounced effects on the archaeal community distribution and methanogenesis pathways.

### Multivariate analysis

**Figure 6:**
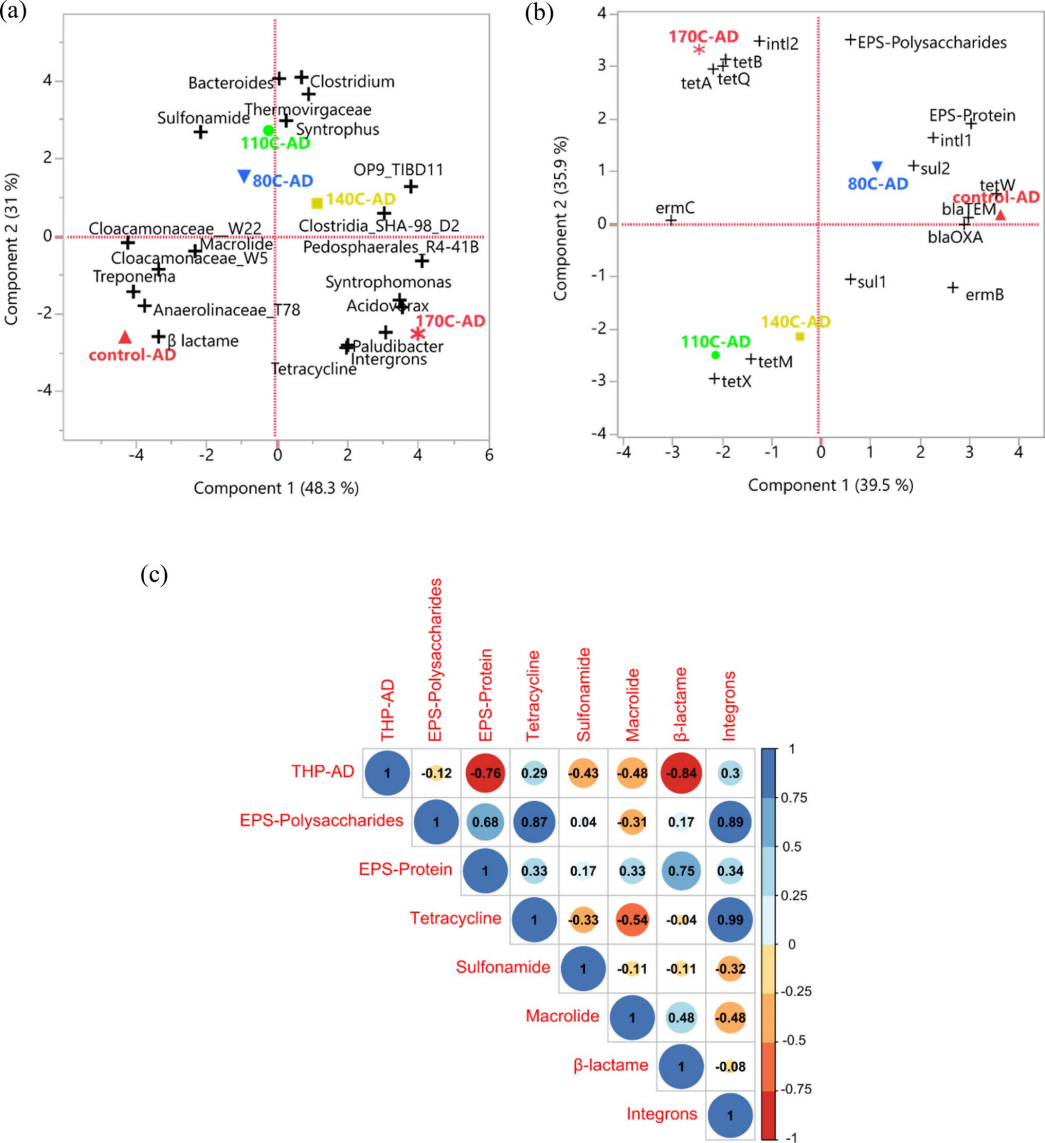
(**a**) PCA of ARGs and microbial communities, (**b**) PCA of ARGs and EPS components, and (**c**) Correlation analysis between EPS components and ARG abundance under different conditions.

The multivariate PCA was performed to evaluate the correlation between ARG abundance and bacterial communities in digested samples (Fig. 6a). For THP-110 °C and THP-80 °C, *Clostridium*, *Bacteroides*, *Thermovirgaceae*, and *Syntrophus* were closely associated with sulfonamide resistance genes. *Cloacamonaceae_W22*, *Cloacamonaceae_W5*, *Treponema*, and *Anaerolinaceae* were clustered in a different quadrant close to the control and associated with β-lactam and macrolide resistance genes. The tetracycline resistance genes, strongly correlated with integrons, were close to THP-170 °C, where *Acidovorax*, *Paludibacter*, and *Syntrophomonas* genera were dominant. Based on previous reports, *Clostridium*, *Bacteroides*, *Treponema*, *Paludibacter*, *Syntrophomonas, Acidovorax* species are potential ARG hosts ^67–70^. For instance, the prevalence of macrolide resistance genes in *Treponema* species has already been widely reported ^68^. Thus, THP played a critical role in shaping the bacterial communities and consequently changed the ARG profiles.

Interestingly, digested THP-80 °C, THP-110 °C, and THP-140 °C samples showed a similar decreasing trend for EPS (discussed earlier) and total ARGs, indicating a possible positive correlation between them. Therefore, the relationships between ARG abundances and EPS composition were further analyzed (Fig. 6b). EPS proteins, *int*l1, *sul2*, *bla*_*TEM*_, *bla*_*OXA*_*,* and *erm*B were located close to the control and THP-80 °C samples. On the other hand, *intl2*, *tet*B, *tet*Q, and *tet*A were associated with EPS polysaccharides and were in a different quadrant close to the THP-170 °C sample. However, *sul*1 was close to the THP-140 °C sample, while *tet*M and *tet*X were located in a different quadrant close to the THP-110 °C sample. However, different functional components of the EPS layers could affect the ARGs abundance. For instance, humic acids have strong adsorption to the DNA molecules by ligand binding, hydrophobic interactions, precipitation, and aggregation^24^. These results indicate that ARG profiles responded differently to the THP operating temperatures. Moreover, different EPS components (polysaccharides and proteins) were correlated with different ARG types/subtypes.

To further understand the relationship between ARG and EPS, correlation analysis was performed. Figure 6c shows the correlation coefficients of THP-AD, EPS components, and ARG abundances in digested sludge. Obviously, there was a positive correlation between the EPS protein component and all variables except for the THP-AD (*r* = − 0.76). In contrast, all the process variables showed a high negative correlation with THP-AD except for the tetracycline resistance genes and integrons (*r* = 0.29 and 0.30), respectively. For instance, a strong positive correlation between the EPS protein component exhibited a strong positive correlation with *β*-lactam resistance genes (*r* = 0.75), while the correlation with other ARGs, such as sulfonamides and macrolides (*r* = 0.17 and 0.33, respectively) was fairly weak. On the other hand, EPS polysaccharides showed a positive correlation with all ARGs, especially with tetracycline resistance genes (*r* = 0.87), while macrolides resistance genes were the only exception (*r* = − 0.31). Also, the integrons, which are considered biomarkers for ARG spread ^71^, exhibited a very strong correlation with tetracycline resistance genes (*r* = 0.99). In contrast, the integrons showed a relatively weak correlation with other ARGs.

### Implications

Our results suggest that different EPS components (proteins and polysaccharides) correlate with different ARGs and MGEs. As an important component of sludge, EPS may provide ample adsorption sites for ARGs and play a critical role in their propagation^23,45,72^. Different sludge EPS components have different functional groups, such as carboxyl, phenolic, hydroxyl, etc.^73^. Thus, the adsorption of different ARGs and MGEs onto various EPS components can be different. Interestingly, the digestate THP-140 °C sample had the lowest level of proteins and polysaccharides among all digestate samples, exhibiting the lowest ARG abundance. Overall, these results infer a functional link between EPS (proteins and polysaccharides) composition and ARGs in AD of thermally hydrolyzed sludge under different temperatures. Although a recent report suggested that EPS-associated ARGs would present a most significant portion of ARGs in sludge^23^, the relationships observed in the multivariate analysis in this study remain correlational. Thus, further research should focus on the detailed characterization and changes of EPS-associated, intracellular, and cell-free ARGs under THP. Also, different layers of EPS might affect the fate and abundance of ARGs^72^, which require further investigations.

## Supplementary Information

Supplementary Information.

## Data Availability

The raw 16S rRNA sequencing data were deposited in the Sequence Read Archive (SRA) of National Center for Biotechnology Information (NCBI) under BioProject accession number: PRJNA810055 (Samples accession number: SAMN26226237).
