# Supplementary material for: Propagation of antibiotic resistance genes during anaerobic digestion of thermally hydrolyzed sludge and their correlation with extracellular polymeric substances
Source: Sci Rep. 2022 Apr 25;12:6749. doi: 10.1038/s41598-022-10764-1 (PMC9038762; doi:10.1038/s41598-022-10764-1)
Supplement: Supplementary file 1 — Supplementary Information. [file 41598_2022_10764_MOESM1_ESM.docx]

**Propagation of antibiotic resistance genes during anaerobic digestion of thermally hydrolyzed sludge and their correlation with extracellular polymeric substances**

Nervana Haffiez, Seyed Mohammad Mirsoleimani Azizi^1^, Basem S. Zakaria^1^, Bipro Ranjan Dhar^*^

Civil and Environmental Engineering, University of Alberta, 116 Street NW, Edmonton, AB T6G 1H9, Canada

^1^Two co-authors contributed equally

^*^Corresponding author. Bipro Ranjan Dhar ([bipro@ualberta.ca](mailto:bipro@ualberta.ca))

- **Number of Tables: 3**
- **Number of Figures: 5**

| **Table S1.** Primers used for studying 16S rRNA and ARGs | | |
| --- | --- | --- |
|  | **Forward (5’-3’)** | **Reverse (5’-3’)** |
| *tet*A | GCTACATCCTGCTTGCCTTC | CATAGATCGCCGTGAAGAGG |
| *tet*B | GGCAGGAAGAATAGCCACTAA | AGCGATCCCACCACCAG |
| *tet*C | GCGGGATATCGTCCATTCCG | GCGTAGAGGATCCACAGGACG |
| *tet*W | GAGAGCCTGCTATATGCCAGC | GGGCGTATCCACAATGTTAAC |
| *tet*M | ACAGAAAGCTTATTATATAAC | TGGCGTGTCTATGATGTTCAC |
| *tet*Q | AGAATCTGCTGTTTGCCAGTG | CGGAGTGTCAATGATATTGCA |
| *tet*X | CAATAATTGGTGGTGGACCC | TTCTTACCTTGGACATCCCG |
| *sul*1 | CGCACCGGAAACATCGCTGCAC | TGAAGTTCCGCCGCAAGGCTCG |
| *sul*2 | TCCGGTGGAGGCCGGTATCTGG | CGGGAATGCCATCTGCCTTGAG |
| *erm*B | GATACCGTTTACGAAATTGG | GAATCGAGACTTGAGTGTGC |
| *erm*C | TTTGAAATCGGCTCAGGAAAA | ATGGTCTATTTCAATGGCAGTTACG |
| *bla*_OXA_ | ATATCTCTACTGTTGCATCTCC | AAACCCTTCAAACCATCC |
| *bla*_TEM_ | ATCAGCAATAAACCAGC | CCCCGAAGAACGTTTTC |
| *intl*1 | CCTCCCGCACGATGATC | TCCACGCATCGTCAGGC |
| *intl*2 | GTTATTTTATTGCTGGGATTAGGC | TTTTACGCTGCTGTATGGTGC |
| 16S rRNA | CCTACGGGNGGCWGCAG | GACTACHVGGGTATCTAATCC |

**Table S2.** Kinetic parameters estimated with the modified Gompertz model.

| Experimental condition | Maximum methane yield, V_m_^*^ (mL/g COD) | Maximum methane production rate, R (mL/g COD/d) | Standard error for R | Lag phase, λ (d) | Standard error for λ |
| --- | --- | --- | --- | --- | --- |
| THP-80 ⁰C-AD | 208.7 | 13.15 | 0.82 | -3.03 | 0.61 |
| THP-110 ⁰C-AD | 245.24 | 16.58 | 0.91 | -1.27 | 0.47 |
| THP-140 ⁰C-AD | 276.3 | 14.24 | 0.83 | 3.53 | 0.62 |
| THP-170 ⁰C-AD | 205 | 10.62 | 0.59 | 8.18 | 0.59 |
| Control-AD | 172.76 | 9.39 | 0.66 | -5.35 | 0.85 |

*V_m_ values were fixed at the experimental total cumulative methane yields

**Fig. S1.** Changes in SCOD/TCOD and VSS/TSS ratios after THP.

(a)

(b)

(c)

**Fig. S2.** (a) Estimated free ammonia nitrogen (FAN) after THP, (b) measured TAN concentrations and pH after BMP test, and (c) estimated FAN after BMP test.

**Changes in functional groups of macromolecules**

FTIR analysis of solids was carried out to identify the effects of THP on functional groups associated with macromolecular compounds. The raw sludge sample showed the highest absorbance peaks. In contrast, the peaks intensity decreased for THP samples, indicating that THP could induce changes in functional groups and chemical structures of macromolecular compounds. The peak in the range of 3200-3600 cm^-1^ indicates the presence of O-H vibration of alcoholic and carboxylic groups in addition to amide hydrogen vibrations ^1^. However, the absorption peaks between 2800-2980 cm^-1^ refer to the stretching of aliphatic C-H bonds, indicating lipid content ^2,3^. For the region of 1300-1700 cm^-1^, the peak at 1600-1700 cm^-1^ is for conjugated C=O and C=C in aromatic compounds, indicating the presence of aldehydes, ketones, proteins, and carboxylic acids, while the peak at 1540 cm^-1^ is for the amide and carboxylate C=O ^4^. Additionally, peaks that appeared in the ranges of 1030-1200 cm^-1^ present the stretching of a single C-O bond in esters, ethers, polysaccharides, and carboxylic acids ^2,3^. Noticeably, all the absorption peaks intensities decreased after the THP. For instance, the remarkable reduction in the peak intensity in the range 2800-2980 cm^-1^ indicates the degradation of lipids and fats after THP. Also, peak intensities at 1600-1700 cm^-1^ are considerably reduced with temperature raising attributed to proteins and other aromatic compounds solubilization. The same decreasing trend in the peak’s intensities at 1000-1050 cm^-1^, indicates carbohydrates degradation. Thus, FTIR results further confirmed solubilization of macromolecular organics after the THP. Moreover, the gradual decrease of the absorption peaks with increasing the THP operating temperature indicates the relationship between temperature and solubilization efficiencies.


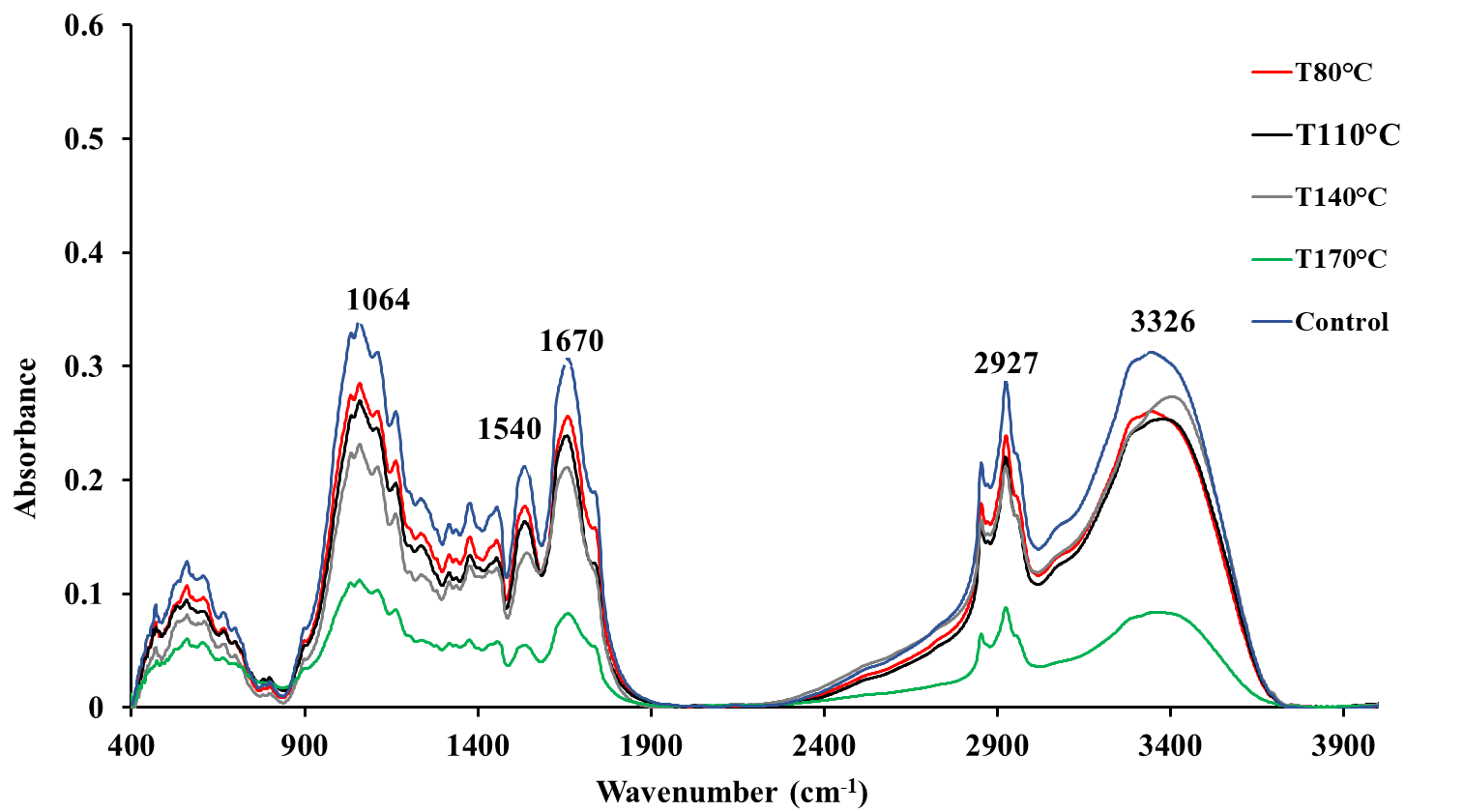


**Fig. S3.** FTIR spectrum of raw and pretreated samples.

**Fig. S4.** Extracellular polymeric substances (polysaccharides and proteins) after AD.

| Table S3. The diversity and richness of the microbial communities. | | | | | |
| --- | --- | --- | --- | --- | --- |
|  | Chao 1 | Pielou | OTUs | Shannon | Coverage |
| Control | 171 | 0.87 | 170 | 6.4 | 1 |
| Control-AD^a^ | 35 | 0.64 | 35 | 3.3 | 1 |
| THP-80^o^C | 179 | 0.83 | 174 | 6.2 | 1 |
| THP-80 ^o^C-AD^a^ | 75 | 0.77 | 74 | 4.8 | 1 |
| THP-110^o^C | 120 | 0.81 | 117 | 5.6 | 1 |
| THP-110^o^C-AD^a^ | 83 | 0.75 | 83 | 4.8 | 1 |
| THP-140^o^C | 126 | 0.81 | 126 | 5.6 | 1 |
| THP-140^o^C-AD^a^ | 91 | 0.78 | 91 | 5.1 | 1 |
| THP-170^o^C | 95 | 0.79 | 95 | 5.2 | 1 |
| THP-170 ^o^C-AD^a^ | 93 | 0.84 | 92 | 5.5 | 1 |

^a^Digestate from BMP test

**Fig. S5.** Relative abundance of bacterial communities at the phylum level

**References**

1. Azizi, S. M. M. *et al.* Low-temperature thermal hydrolysis for anaerobic digestion facility in wastewater treatment plant with primary sludge fermentation. *Chem. Eng. J.* **426**, 130485 (2021).

2. Castaldi, P., Alberti, G., Merella, R. & Melis, P. Study of the organic matter evolution during municipal solid waste composting aimed at identifying suitable parameters for the evaluation of compost maturity. *Waste Manag.* **25**, 209–213 (2005).

3. Chowdhury, B. *et al.* Enhanced biomethane recovery from fat, oil, and grease through co-digestion with food waste and addition of conductive materials. *Chemosphere* **236**, 124362 (2019).

4. Ramesh, A., Lee, D. J. & Hong, S. G. Soluble microbial products (SMP) and soluble extracellular polymeric substances (EPS) from wastewater sludge. *Appl. Microbiol. Biotechnol.* **73**, 219–225 (2006).
